# Prognostic role of homeostasis model assessment and oral glucose tolerance test in nondiabetic patients with Bell’s palsy

**DOI:** 10.3906/sag-1901-151

**Published:** 2020-04-09

**Authors:** Tuğba KARAGÖZ, Ömer BAYIR, Emel ÇADALLI TATAR, Erman ÇAKAL, Ali ÖZDEK, Kemal KESEROĞLU, Mustafa ŞAHİN, Mehmet Hakan KORKMAZ

**Affiliations:** 1 Otorhinolaryngology and Head and Neck Surgery Clinic, Kaman State Hospital, Kırşehir Turkey; 2 Department of Otorhinolaryngology and Head and Neck Surgery, Dıskapı Yıldırım Beyazıt Research andTraining Hospital, Ministry of Health, Ankara Turkey; 3 Department of Endocrinology and Metabolism, Dıskapı Yıldırım Beyazıt Research and Training Hospital,Ministry of Health, Ankara Turkey; 4 Department of Otolaryngology and Head and Neck Surgery, Adnan Menderes University Medical School, Aydın Turkey; 5 Department of Otolaryngology and Head and Neck Surgery, Yıldırım Beyazıt University Faculty of Medicine, Ankara Turkey

**Keywords:** Bell’s palsy, insulin resistance, prediabetes, HOMA-IR index

## Abstract

**Background/aim:**

We aimed to reveal the incidence and predictive role of insulin resistance and distorted oral glucose tolerance test in nondiabetic patients with Bell’s Palsy (BP).

**Materials and methods:**

Eighty-six patients with BP and 28 control subjects; all with normal blood glucose levels and no history of diabetes, were enrolled in the study. We investigated insulin resistance (IR) in all subjects, in terms of HOMA-IR greater than 2.7. Sixty-two of the patients also underwent an oral glucose tolerance test (OGTT).

**Results:**

The mean HOMA-IR value was significantly increased in patients, compared to the control group (3.2 vs 1.6; P < 0.01). IR was detected more in BP patients than in controls (P < 0.05). The patients with higher HOMA-IR values had more severe facial dysfunction at the initial presentation and complete recovery time took longer than the patients with normal HOMA-IR value (75 days vs 42 days; P < 0.05). Following a 2h-OGTT, impaired glucose tolerance and newly diagnosed DM were found in 60% of the patients. Recovery time was significantly longer in prediabetics and newly diagnosed diabetic patients than in patients with normal glycemia (68 days, 52 days, and 32 days, respectively; P < 0.01).

**Conclusion:**

There is a strong linkage between HOMA-IR value and BP prognosis so HOMA-IR value may have a significant role of predicting BP prognosis at presentation.

## 1. Introduction

Facial mimetic muscles are a group of striated skeletal muscles innervated by the facial nerve (FN) with a complex range of functions including the expression of the emotional status involuntarily. FN is also composed of parasympathetic to the lacrimal, submandibular and sublingual glands; sensory innervation to the part of the external ear and contribution to taste at the anterior two thirds of tongue [1]. Due to its complex and bony covered anatomical course, FN is the most prone nerve to ischemic and inflammatory injuries among the all cranial nerves [2]. The ancients suffered so much from facial paralysis than any other types of cranial neuropathies that it has become one of the most significant clinical problems throughout history even in the riddle of Leonardo da Vinci’s ‘Mona Lisa’ [3]. Facial paralysis has a broad range of possible aetiologies including numerous acquired and some congenital causes. Bell’s palsy (BP), defined as idiopathic facial paralysis, is the most frequent type with an incidence of 20/100.000 in the Western adult population [4].

There is no identified cause of BP*, *but some possible aetiologies include viral inflammation and/or micro*-*vascular problems. The prognosis for individuals with BP is generally very good, but diabetes associated microangiopathy may be a potential etiologic and poor prognostic factor in BP [5]. Microcirculatory failure of the vasa nervosum impairs nerve metabolism and causes venous stasis*, *resulting in accumulation of toxic metabolites accompanied by a cascade of oedema and ischemia [6].

On the other hand, neuropathy is the most common chronic complication of diabetes mellitus*. *There are 3 types of diabetic neuropathy: autonomic neuropathy, peripheral neuropathy, mononeuropathy [7]. Metabolic factors play role in autonomic and peripheral neuropathy, whereas involvement of vasa nervorum leads to the mononeuropathy [7]. Subclinical facial nerve dysfunctions were detected in 70% of patients with diabetic polyneuropathy [8]. Early stages of hyperglycaemia even cause distal peripheral nerve damage [8]. Impaired glucose tolerance (IGT) was observed in 45% of 187 patients with idiopathic neuropathies [9].

Neuropathic pain is one of the most common symptoms of IGT [10]. Prevalence of glucose metabolism disorders and insulin resistance were found higher in nondiabetic patients with BP than control group [11].

Prediabetes, defined as impaired fasting glycemia (IFG) and IGT associated with insulin resistance (IR), have an increased risk of developing diabetes [12]. Mechanisms underlying the pathogenesis of diabetic or even prediabetic neuropathy is associated with IR leading to neuronal injury via neuronal insensitivity to neurotropic properties of insulin [13].

In this clinical study; we evaluated HOMA-IR, and prediabetic status with an oral glucose tolerance test (OGTT) in nondiabetic patients with BP. Our purpose was to reveal the clinical relationship between BP and IR/prediabetes. We also assessed the effect of higher HOMA-IR value and prediabetes status on the prognosis and recovery period of BP*.*

## 2. Materials and methods

The study was approved by the Ethical Committee of Ministry of Health, Dışkapı Training and Research Hospital on March 2014 (all patients and people in control group gave their consent and informed consent was obtained and signed by each, in accordance with the official standards of the Declaration of Helsinki (1964), local laws and regulations). The registration number was 14/34.

Between March 2014 and March 2015, we studied prospectively 86 nondiabetic patients with Bell’s palsy (40 women and 46 men) and 28 nondiabetic control subjects (18 women and 10 men) without a history of facial nerve palsy. All the subjects had normal blood glucose levels (fasting level < 100 gr/dL).

All the patients and the controls underwent the following tests: pure tone audiogram, stapes reflex, blood chemistry screen (including Vit B12, folic acid, serum HbA1c, cholesterol, triglyceride, iron and uric acid levels), temporal bone magnetic resonance imaging (MRI).

According to the World Health Organization classification, a body mass index (BMI) over 25 kg/m2 was defined as overweight, and over 30 kg/m2 as obese [14].

Exclusion criteria included the followings; previous otologic disease history and surgery, hereditary hearing loss, congenital facial paralysis, pregnancy, autoimmune rheumatic diseases, head trauma, history of alcohol abuse, chronic liver/kidney disease, the use of medications that affect glucose metabolism (such as beta-blockers, corticosteroids).

The House-Brackmann (HB) scale was used to quantify and describe facial nerve dysfunction.

To find a more accurate prognostic factor as well as to yield an estimate of insulin resistance in nondiabetic falsy palsy patients we measured the HOMA-IR measured by fasting blood glucose and insulin levels in 86 patients before starting steroid treatment. HOMA-IR used for evaluation of insulin sensitivity was calculated by the formula; basal plasma glucose (mg⁄dL) × basal plasma insulin (UI ⁄mL) ⁄ 405. HOMA-IR over 2.7 was admitted as abnormal according to the study of Bosco et al. and a study of Turkish population [11,15]. Written informed consent was obtained from 62 of 86 patients before performing OGTT. A zero time (fasting) venous blood sample was drawn after 10 h of overnight fast. The patients were then given a 75 g oral glucose load within a 5 min time frame. Venous blood was drawn after an interval of 2 h for measurement of glucose [16]. Systemic administration of corticosteroids was used for treatment of BP patients as soon as OGTT was performed and HOMA-IR was calculated. The standard treatment regimen consisted of initial administration of intravenous methylprednisolone 250 mg. On day 2, patients received oral Metilprednizolon 1 mg/kg/day. The corticosteroid dose was tapered 20 mg every second day until termination. 

## 3. Results

This study included 86 nondiabetic patients with BP; 40 were females (46%) and 46 were males (54%) and their ages ranged from 18 to 75 years with a mean of 41. Of the 28 control subjects; 18 were females (65%) and 10 were males (35%) and their ages ranged from 18 to 70 years, with a mean age of 38. None of the controls had a history of diabetes and BP. No significant difference was found in age and sex between 2 groups (P > 0.05) (post hoc test).

Mean BMI of 86 patients was found as 28 kg/m2; they were divided into 2 groups 17 patients had normal weight (BMI < 25), and 67 patients were overweight and obese. The mean BMI of the control group was 27.3 kg/m2; BMI less than 25 kg*/*m2 was found in 7 subjects while BMI ≥ 25 kg/m2 was observed in 21 subjects. No significant difference was found between BMI distributions of the 2 groups (P > 0.05) (t test).

Four of 86 patients already had hypertension; but others had no history of systemic disease. Similarly, 2 of 28 control subjects had hypertension. The mean time between disease onset and diagnosis was 1.8 day (min 1 day, max 10 days; 80 of 86 patients applied within 3 days of clinical onset). BP affected the left side in 59% of the patients. Twelve patients suffered from previous episodes of unilateral facial nerve paralysis; 74 patients had their first attack.

Most of the patients suffered from incomplete facial palsy at the initial diagnosis (Table 1).

**Table 1:** Distribution of clinical severity of 86 patients with facial palsy at the initial diagnosis.

House Brackmann grading scale	n = 86 Count (%)
2	35 (41.4%)
3	21 (24.1%)
4	16 (18.4%)
5	6 (6.9%)
6	8 (9.2%)

The mean value of HOMA-IR in patients’ group was found as 3.2 and was higher than the mean value of HOMA-IR in the control group which was calculated as 1.6. 

Therefore, the mean HOMA-IR of the patients were significantly higher than the controls (P < 0.001) (Mann–Whitney test).

Of the 86 patients; 48 (55.8%) had HOMA-IR of <2.7; and 38 (44.2%) had HOMA-IR of ≥2.7; and of the 28 control subjects 23 (82%) had HOMA-IR of <2.7; and 5 (18%) had HOMA-IR of ≥2.7 (P < 0.05) (Pearson chi-square test) (Table 2). The percentage of high HOMA-IR subjects were higher in the BP group when compared to the controls.

**Table 2:** Distribution of patients and control groups in terms of their HOMA-IR index with cut off value of 2.7.

Groups	Number of patients		Total
	HOMA-IR value of <2.7	HOMA-IRvalue of ≥ 2.7	
Bell’s palsy patients	48 (55.8%)	38 (44.2%)	86 (100.0%)
Control	23 (82.1%)	5 (17.9%)	28 (100.0%)

P: 0.023

Average value of HbA1c in patients’ group was found as 5.6 and 5.4 in the control group, the difference was not significant (P > 0.05).

OGTT could be performed in 62 of the patients and following the 2 h-OGTT, we found an abnormal glucose metabolism in 37 patients (29 patients with IGT and 8 patients with new onset DM). 

We could determine the exact recovery time of 56/86 patients. Recovery time of patients with HOMA-IR value of ≥2.7 was significantly longer than patients with HOMA-IR value of <2.7 (75.8 days and 42.9 days, respectively; P < 0.048; Mann–Whitney U) Recovery time, with respect to OGTT, was statistically longer in prediabetic and newly diagnosed diabetic patients than in patients with normal glycaemia (68 days, 52, days and 32 days, respectively, P < 0.01). 

When we looked at the relationship between HOMA-IR and HB stage at the initial patient presentation; the group with lower HOMA-IR value had statistically more patients with HB grade 2 and 3. Additionally 18 of the 30 patients with HB grade 4,5, and 6 were detected in the higher HOMA-IR group (P < 0.05; Pearson chi-square) (Table 3).

**Table 3:** Relationship between HOMA-IR and HB stage at presentation.

		Number of patients	
		HOMA-IRvalue of <2.7	HOMA-IRvalue of ≥2.7
House Brackman grading scale	2	26 (74.3%)	9 (25.7%)
	3	10 (47.6%)	11 (52.4%)	
	4-5-6	12 (40%)	18 (60%)

P = 0.015 (chi-square test)

## 4. Discussion

Bell’s palsy as an acute impairment of facial nerve functions, is a diagnosis of exclusion and affects men and women equally. The peak incidence is between the ages of 15–45, but may occur at any age [2]. There is no known blood test to give the diagnosis of BP or to predict the prognosis. There is also no proved etiologic factor or disease in BP. Viral infections and diabetes are considered to have some role in the etiopathogenesis and prognosis of BP. Our hypothesis in the present clinical study was; IR or prediabetes may play a role in the clinical severity and prognosis of the disease in nondiabetic BP patients. Therefore, we included the BP patients with normal fasting blood glucose levels (<100 gr/dL) in this study. All our patients were given steroids but no antiviral agents, all our patients recovered to at least HB grade 2 with different recovery periods, none of our patients underwent surgery for facial nerve decompression. In our study considering the optimal HOMA-IR cut off value as 2.7 for Turkish population, 45% of BP patients presented with higher HOMA-IR levels, as well as the 18% of the controls. In addition to that, the mean value of HOMA-IR was 3.2 in the patients’ group, whereas it was 1.6 in the control group. Our results are consistent with the hypothesis that insulin resistance is more frequent in nondiabetic BP patients. These data may point out to a role for insulin resistance in the etiopathogenesis of BP as well.

The mean age of the patients in our study was 41, parallel to that of the literature. While 30% of BP cases reported in the literature suffer from partial paralysis at the onset, 85% of our patients suffered from partial paralysis (House Brackman grade 2-3-4). In our study, in the lower HOMA-IR group (<2.7) there were less patients presenting with severe HB grades (5–6) but more with lower HB grades (2–3–4). There were totally 14 patients presenting with HB grade 5 and 6 in the study, and of these patients 10 had HOMA-IRof >2.7. Our findings suggest that, those BP patients with higher HB grades had higher HOMA-IR. Therefore it is possible to comment that prediabetic status or IR can be associated with worse HB grade at presentation. This result may also be consistent with the hypothesis that IR can have a role in the etiopathogenesis of BP.

Based on the OGTT in 62 of the BP patients, prevalence of glucose metabolism abnormalities was found in 60% of our patients (47% with IGT and IFG and 13% with a newly diagnosed DM). BMI over 25 kg/m2 was found in 77% of patients and 75% of control group. This data emphasizes that HOMA-IR value and/or impaired OGTT, rather than BMI may play role in BP. Our study may have some criticisms. Firstly the 2h-OGTT was not performed after loading the diet with carbohydrates for 4 days, this is because we should start steroid treatment as soon as possible and secondly, we could not perform OGTT to control group because it was time consuming, costly, and risky attempt.

BP is considered to be a consequence of entrapment neuropathy following inflammation, oedema and strangulation due to microangiopathies, which responds to corticosteroids significantly [6,17]. Unlike other cranial nerves, facial nerve with its long bony canal is open to effects of inflammation and oedema and presents with various degrees of nerve damage. Perineural vascular congestion gives rise to nerve injury determined on MRI and detected perioperatively while improvement was also shown after steroid treatment [17,18,19]. Considering that DM is frequent in BP patients, Adour et al. advised screening for blood sugar elevation for those, who have recurrent facial paralysis [20]. On the other hand, Schwann cells and the myelin sheath are more likely to be affected in diabetics than nondiabetic patients [20]. The early stages of hyperglycaemia are critical for peripheral nerve damage which may result in peripheral neuropathy [8,9]. Prediabetic neuropathy affects sensory nerve fibres particularly more than motor nerve fibres [21].

Osmosis in early stages of hyperglycaemia directly causes myelin sheath damage in small nerve fibres [22]. Therefore, prediabetic neuropathy is an indicative of early distal small sensory neuropathy whereas diabetic neuropathy causes loss of vibration sense [23].

Even though some studies could bring a conclusion that DM or hyperglycaemia itself does not cause facial nerve palsy but may facilitate the demyelination and degeneration of the facial nerve during HSV-1 infection [24]. This may end with facial nerve paralysis, and histopathologically confirmed that HSV-1 infection and nerve degeneration were more remarkable in the DM mice than in non-DM mice [24].

Sittel et al. reported no significant differences in complete recovery rate from BP, between diabetic and nondiabetic groups [25]. The underlying reason for that may be the possible presence of prediabetic and IR patients in nondiabetic group. In our study, all our patients recovered to at least HB grade 2 regardless of the prediabetic status or IR. However, our patients with HOMA-IR of >2.7 had longer recovery time compared to those with HOMA-IR of <2.7 (76 days vs 43 days, respectively). Although there was no apparent hyperglycaemia, higher HOMA-IR had some role in the recovery period time. It seems that neuropathy in BP is not only correlated to hyperglycaemia associated pathogenesis. Prediabetic neuropathy has been delineated by multiple potential pathogenical mechanisms. Leading to oxidative stress in addition to activation of protein kinase C (PKC) and polyol pathway, hyperglycaemia has neurotoxic effect directly [26]. In an animal study, hyperglycaemia induces apoptotic changes in dorsal root ganglion neurons and Schwann cells which result in damaged mitochondria in the nerve fibre and premature neuronal death [26,27,28]. In vivo acute transient hyperglycaemia episodes lead to endothelial dysfunction due to increase of reactive oxygen metabolites. This may result in neuronal metabolic dysfunction and consequent direct DNA damage which promote neuronal cell death [29]. In diabetic patient’s chronic hyperinsulinemia may cause reduction in endoneural oxygen, reduced blood flow, and epineurial arteriovenous shunting and a compensatory response to endoneural ischemia/hypoxia; and these may all contribute to chronic nerve ischemia [6].

The relationship between facial palsy and DM is well known. However, there are few controlled studies on the association of facial palsy and manifestation of prediabetes and insulin resistance. The most widely accepted prognostic indicator of BP is electroneuronography (ENoG) test, however we believe that calculating HOMA-IR value may also give additional clues to predict the prognosis. In a clinical study it was stated that; although being diabetic does not influence the severity of facial palsy at the time of onset, recovery time from Bell’s palsy took longer in patients with diabetes compared to the nondiabetic patients [30]. Our data suggested that patients with higher HOMA-IR presented with higher HB grade and they recovered later than the lower HOMA-IR patients. Hyperglycaemia induces direct nerve injury by similar pathogenetic mechanisms either in IGT or in diabetes [21]. Glycaemic control is a crucial factor in the progress of neuropathy [31]. Therefore, targeted therapy in BP may also include regulation of glucose metabolism even in nondiabetic patients with IR. 

Our clinical data documented that there may be a linkage between facial palsy and insulin resistance, in terms of severity and prognosis in Bell’s palsy. We found that in nondiabetic patients with BP; those with higher HOMA IR of >2.7 and prediabetics had longer recovery period. Therefore, further studies in larger groups are needed in understanding the underlying potential mechanisms of insulin resistance and distorted OGTT.

## Acknowledgments

Each of the authors has contributed to, read and approved this manuscript. This paper was presented at ENT WORLD CONGRESS IFOS, Paris on 27 June 2017. The authors thank Sevilay Karahan (Department of Biostatistics, Hacettepe University School of Medicine, Ankara, Turkey) for helping to the statistical analysis. 

## Informed Consent

The study was approved by the Ethical Committee of Ministry of Health, Dışkapı Training and Research Hospital on March 2014 (all patients and people in control group gave their consent and informed consent was obtained and signed by each, in accordance with the official standards of the Declaration of Helsinki (1964), local laws and regulations). The registration number was 14/34.
